# Control of optical spin Hall shift in phase-discontinuity metasurface by weak value measurement post-selection

**DOI:** 10.1038/srep13900

**Published:** 2015-09-10

**Authors:** Y.U. Lee, J.W. Wu

**Affiliations:** 1 Department of Physics and Quantum Metamaterials Research Center, Ewha Womans University, Seoul, 03760, Korea

## Abstract

Spin Hall effect of light is a spin-dependent transverse shift of optical beam propagating along a curved trajectory, where the refractive index gradient plays a role of the electric field in spin Hall effect of solid-state systems. In order to observe optical spin Hall shift in a refraction taking place at air-glass interface, an amplification technique was necessary such as quantum weak measurement. In phase-discontinuity metasurface (PMS) a rapid phase-change along metasurface takes place over subwavelength distance, which leads to a large refractive index gradient for refraction beam enabling a direct detection of optical spin Hall shift without amplification. Here, we identify that the relative optical spin Hall shift depends on incidence angle at PMS, and demonstrate a control of optical spin Hall shift by constructing weak value measurement with a variable phase retardance in the post-selection. Capability of optical spin Hall shift control permits a tunable precision metrology applicable to nanoscale photonics such as angular momentum transfer and sensing.

According to Maxwell’s description, the transversality is a fundamental property of electromagnetic wave. In an optical beam propagating along a curved trajectory, the transversality results in spin-orbit interaction, which is one example of interaction Hamiltonians coupling slow and fast systems. A coupling of slow and fast systems leads to reciprocal effects of action and reaction between the two systems, which are described coherently in terms of the Berry phase and curvature^1^,^2^. In addition, the presence of a degenerate point in the energy-momentum dispersion relation of light allows an introduction of topological magnetic monopole in describing spin-orbit interaction^3^,^4^.

In spin-orbit interaction of an optical beam along a curved trajectory, the beam trajectory and optical spin correspond to the slow and fast systems, respectively. Polarization-plane rotation of light along a coiled optical fiber results from the effect of a curved beam trajectory (slow) on optical spin (fast), which is a manifestation of the Berry phase in the light polarization^5^. On the other hand, the effect of optical spin (fast) on a curved beam trajectory (slow) gives rise to a spin-dependent transverse shift of optical beam centroid, i.e., spin Hall effect of light (SHEL)^6^,^7^. The beam trajectory is described by the Lorentz force in momentum space, 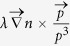, where 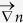 is the refractive index gradient and 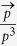 is the topological magnetic monopole Berry curvature associated with optical beam of spin *λ*^8^,^9^,^10^.

In air-glass interface, the magnitude of 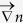 is not large enough, and in order to obtain an image showing optical spin Hall transverse shift, it was necessary to adopt a multiplying prism to have multiple total internal reflections^7^. In the case of refraction at air-glass interface, a direct detection of the spin-dependent transverse shift was not readily feasible, and a weak measurement amplification technique was adopted for observation, where a nearly crossed polarizer/analyzer is employed to amplify optical spin Hall shift by means of a quantum weak measurement^11^,^12^.

Direct observation of optical spin Hall shift in far-field has been realized in artificial optical structures such as an array of plasmonic rectangular apertures and dielectric gradient metasurfaces^13^,^14^. In contrast, in a phase-discontinuity metasurface (PMS) composed of an array of V-shaped antennas, a rapid phase-change along metasurface over subwavelength distance leads to a large refractive index gradient for cross-polarized scattering lights^15^. The magnitude of SHEL transverse shift is in the order of a few hundreds nanometers at the near IR spectral range, which was directly detected without resorting to a weak measurement amplification technique^16^.

One distinct feature of PMS is that the refractive index gradient is tangential to the metasurface, differently from air-glass interface where the refractive index gradient is normal to the interface. At air-glass interface shown in Fig. 1(a), the radii of circular equifrequency contour are different in air and glass, and transverse shifts cancel out at top and bottom parallel interfaces possessing opposite refractive index gradients^11^. At PMS on a glass substrate, in contrast, the net transverse shift comes from the refractive index gradient of metasurface as shown in Fig. 1(b) with a single circular equifrequency contour with the radius specified by the energy-momentum of light in air.

In this article, first we derive an expression of optical spin Hall transverse shift in PMS. Since the refractive index gradient is tangential to metasurface, rotational symmetry with respect to the surface normal is broken and conservation of total angular momentum does not hold for an optical beam passing through PMS. However, an analysis based on the Berry connection allows for an analytic expression of optical spin Hall transverse shift. Next we show both theoretically and experimentally that the sign of relative transverse shift depends on incidence angle which is understood in terms of an analytic expression of optical spin Hall transverse shift as well as the Berry curvature. Then, we introduce a weak value measurement to control the sign and magnitude of transverse shift by manipulating optical phase retardance in the post-selection. Finally, we demonstrate dynamic control of transverse shift by varying an electric voltage applied to the liquid crystal variable retarder.

## Results

### Optical spin Hall shift in PMS

**Figure 2:**
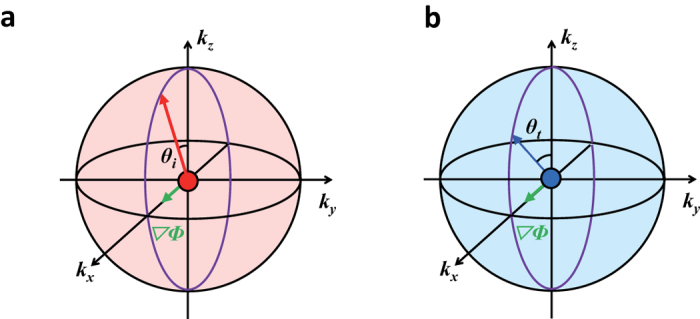
Incidence and refraction wavevectors and phase gradient 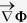 in sphere of equifrequency surface. (**a**) Incidence wavevector (red) and phase gradient 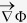 (green) are shown in sphere of equifrequency surface. (**b**) Refraction wavevector (blue) and phase gradient 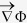 (green) are shown in sphere of equifrequency surface. In both (**a**,**b**) the topological magnetic monopole is located at the center of sphere of equifrequency surface. The Berry curvatures are directed along radial vectors with polar angles determined by incidence and refraction angles *θ*_*i*_ and *θ*_*t*_.

In the equifrequency surface of PMS shown in Fig. 2 the refractive index gradient is along *x*-axis, and the topological magnetic monopole Berry curvatures are radial vectors with directions determined by incidence and refraction angles *θ*_*i*_ and *θ*_*t*_. Transverse shift *δy* upon refraction at PMS is related to the phase gradient 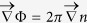 and the Berry connections of incidence and refraction beams, yielding an expression of transverse shift:^9^,^17^


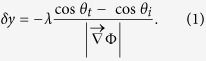


See Supplementary Eq. S2 for derivation of Eq. (1). From the two facts that both positive and negative refractions can take place at PMS and that 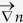 is tangential to PMS surface, the sign and magnitude of transverse shift *δy* depend on incidence and refraction angles *θ*_*i*_ and *θ*_*t*_ as well as 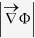, as can be read-off from Fig. 2 and Eq. (1).

**Figure 3:**
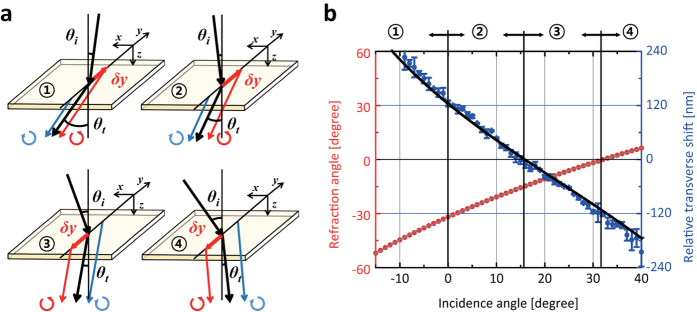
Schematics of refraction and transverse shift. (**a**) Schematics of the beam refraction from air to ① a positive-refraction-low-index medium, ② a negative-refraction-low-index medium, ③ a negative-refraction-high-index medium, and ④ a positive-refraction-high-index medium are shown. (**b**) Theoretical calculation (solid curve) and experimental measurement (solid circle) of refraction angle (red) *θ*_*t*_ and relative transverse shift (blue) are plotted as a function of incidence angle *θ*_*i*_.

Figure 3(a) shows examples how the relative transverse shift of optical beams with spins ±1 changes sign in detail. For *λ* = +1 corresponding to the red arrows in Fig. 3(a), when *θ*_*i*_ < *θ*_*t*_ a positive transverse shift (*δy* > 0) takes place in both positive (①) and negative (②) refractions, and when *θ*_*i*_ > *θ*_*t*_ a negative transverse shift (*δy* < 0) takes place in both negative (③) and positive (④) refractions. In Fig. 3(b) are plotted theoretical calculation (solid curves) and experimental measurement (solid circles) of refraction angle *θ*_*t*_ and transverse shift *δy* as a function of incidence angle *θ*_*i*_.

### Weak value of optical spin Hall shift post-selected with a phase retardance

Weak measurement amplification technique enabled an observation of optical spin Hall shift in air-glass interface^11^. By preparing a polarizer as pre-selection, the weak value is measured by a strong measurement with a nearly cross-polarized analyzer as the post-selection^18^. In PMS, on the other hand, it is not necessary to adopt weak measurement amplification technique for an observation of optical spin Hall shift. However, when it is attempted to control the transverse shift in PMS by an optical means, a weak value measurement can be utilized with a variable phase retardance in the post-selection.

**Figure 4:**
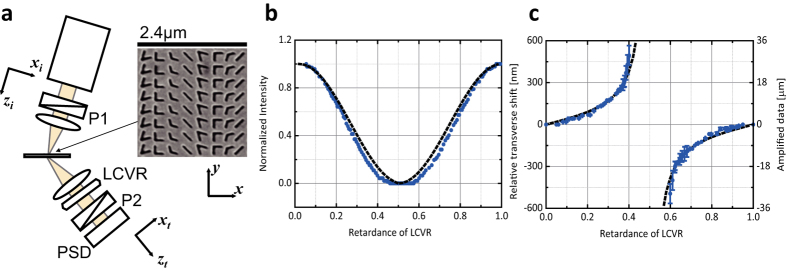
Experimental setup and weak value measurement. (**a**) Schematics of weak measurement with a variable retardance is shown with *P*_1_ = (1, 0)^*T*^ and *P*_2_ = (0, 1) along with SEM image of Babinet complementary phase-gradient metasurface^26^. LCVR is liquid-crystal variable retarder and PSD is a quadrant position sensitive detector. See Methods for the detailed description of sample and measurement. (**b**) Light intensity transmitted through a cross polarizer/analyzer setup is plotted as a function of retardance Γ of LCVR. (**c**) Weak value of optical spin Hall shift post-selected with phase retardance is plotted as a function of retardance Γ of LCVR with the corresponding transverse shift. Blue solid circles are data point and dashed curves are from theoretical calculation.

Optical spin Hall shift is one example of classical analogues of a quantum measurement of the polarization state of a paraxial beam by its transverse amplitude distribution^19^. By introducing a variable optical phase retardance in the post-selection, we can tune the post-selection state 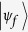 across the whole range of retardance, [0, *π*/2], to control optical spin Hall shift, which is made possible in PMS since optical spin Hall shift is large enough to be detected in the optical far field. We place a phase retarder with variable retardance Γ (modulus of *π*) inside a cross polarizer/analyzer (*P*_1_/*P*_2_) setup in order to control optical spin Hall shift in the weak measurement as shown in Fig. 4(a), where the post-selection state is 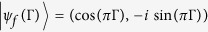.

When optical spin Hall transverse shift is measured at the propagation distance *z* of a Gaussian beam with Rayleigh range of *z*_0_, the observable *metaSHEL* is expressed in terms of the Pauli matrix 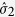 in the linear polarization bases:^17^,^20^,^21^


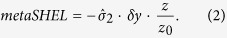


The weak value of transverse shift, post-selected at a retardance Γ, is readily obtained.


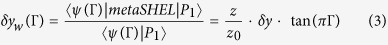


Note that the phase retardance 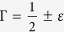 (0 < *ε* ≪ 1) is the range where a weak measurement amplification is achieved. In Fig. 4(b) is plotted the transmitted light intensity through the cross polarizer/analyzer setup of Fig. 4(a) as a function of retardance Γ. Figure 4(c) shows the weak value, *δy*_*w*_(Γ), of an optical beam normally incident on PMS as a function of retardance Γ along with the corresponding transverse shift *δy*. At Γ = 1/4 the weak value *δy*_*w*_(Γ = 1/4) = 7.44*μm*, which corresponds to the transverse shift *δy* = 124 *nm* in the absence of a cross-polarized polarizer/analyzer setup. It is important to note that the phase-retardance dependent weak value is measured in the optical far field^22^,^23^,^24^.

### Images of spin-dependent optical spin Hall shifts

**Figure 5:**
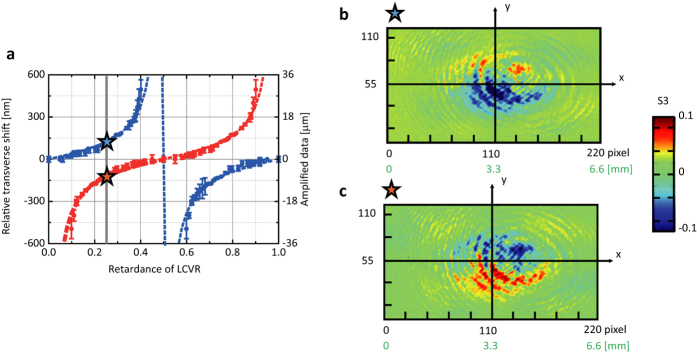
Images of spin-dependent optical spin Hall shifts. (**a**) Relative transverse shifts are measured as a function of retardance Γ in cross-polarized polarizer/analyzer setup (blue solid circles) and in parallel-polarized polarizer/analyzer setup (red solid circles). Images of spin-dependent optical spin Hall shifts at Γ = 1/4 (vertical gray straight line in (**a**)) are obtained by processing each pixel signals in InGaAs-based NIR camera for (**b**) cross-polarized polarizer/analyzer setup, *P*_1_ = (1, 0)^*T*^ and *P*_2_ = (0, 1), and (**c**) parallel-polarized polarizer/analyzer setup, *P*_1_ = (0, 1)^*T*^ and *P*_2_ = (0, 1).

In order to obtain images of spin-dependent optical spin Hall shifts we employed InGaAs-based NIR camera. After two separate measurements of 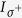 and 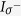, we calculated 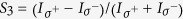 from each pixel signals. We examined how optical spin Hall shift behaves for *s*-polarization (*y*-polarization) and *p*-polarization (*x*-polarization) of extraordinary refraction beam. In Fig. 5(a) blue and red solid circles correspond to *s*-polarization (*y*-polarization) and *p*-polarization (*x*-polarization), respectively. As shown in Fig. 5(b,c), the relative transverse shifts show a sign reversal with the same magnitude, which is different from those observed in air-glass interface.

### Control of optical spin Hall transverse shift by weak measurement post-selection

**Figure 6:**
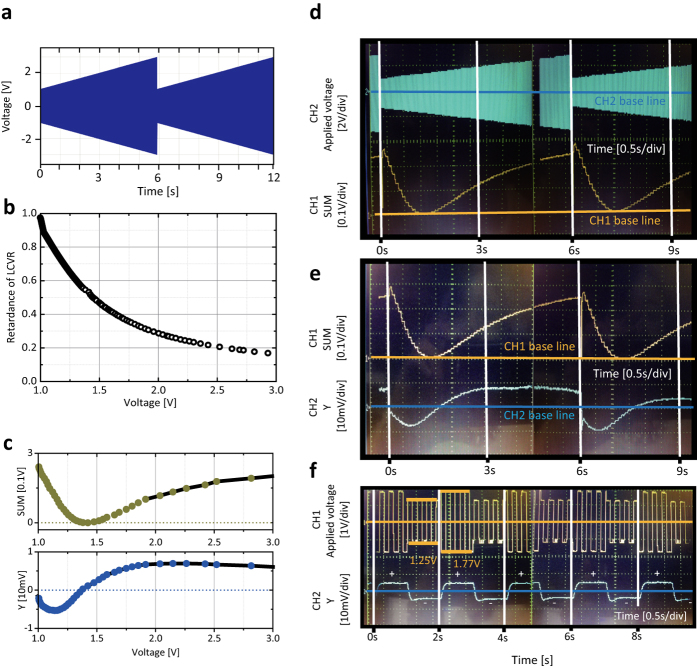
Dynamic control of transverse shift. For a normally incident light in cross polarizer/analyzer setup optical spin Hall transverse shift is dynamically controlled. (**a**) Saw-tooth waveform of LCVR driving voltage is plotted. (**b**) Retardance of LCVR and (**c**) SUM and Y of PSD are plotted as a function of LCVR driving voltage. Oscilloscope traces of (**d**) saw-tooth waveform and SUM of PSD and (**e**) SUM and Y of PSD are shown. (**f**) Switching between positive and negative Y is demonstrated as driving voltage is varied.

Since the weak value is post-selected at phase retardance Γ, an electric manipulation of phase retardance in LCVR allows a control of the weak value *δy*_*w*_. A saw-tooth waveform of LCVR driving voltage is programmed as shown in Fig. 6(a) to obtain a time-varying phase retardation, and Fig. 6(b) is a plot of the measured phase retardance of LCVR as a function of LCVR driving voltage. On the top panel of Fig. 6(c) is re-plotted the transmitted light intensity in Fig. 4(b) as a function of LCVR driving voltage, corresponding to the value of SUM = *q*_1_ + *q*_2_ + *q*_3_ + *q*_4_ of a position-sensitive detector (PSD). On the bottom panel of Fig. 6(c) is plotted the product of the transmitted light intensity in Fig. 4(b) and the relative transverse shift in Fig. 4(c) as a function of LCVR driving voltage, corresponding to the value of Y = (*q*_1_ + *q*_2_) − (*q*_3_ + *q*_4_) of the PSD, associated with optical spin Hall shift. Here, *q*_1_, *q*_2_, *q*_3_, and *q*_4_ represent upper left, upper right, lower left, and lower right quadrant of the PSD.

In order to demonstrate a dynamic control of transverse shift, we monitored SUM and Y from the PSD by oscilloscope, where a saw-tooth waveform of LCVR driving voltage is adopted with 1.0 *V* and 3.0 *V* as the initial and final voltages, covering the phase retardance from 0 to 1 (modulus of *π*). Dual oscilloscope traces of a saw-tooth waveform of LCVR driving voltage (channel 2) and SUM (channel 1) are shown in Fig. 6(d), and dual oscilloscope traces of SUM (channel 1) and Y (channel 2) are shown in Fig. 6(e).

As can be seen in Fig. 6(e), there occurs a sign reversal in Y (channel 2) at LCVR driving voltage of 1.45 *V* corresponding to 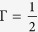, in the vicinity of which a weak measurement amplification is achieved. This leads to a switching behavior of post-selected optical spin Hall transverse shift when the phase retardance is varied crossing 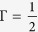. Furthermore, the sign and magnitude of optical spin Hall shift is precisely controllable by manipulating phase retardation at a given incidence angle. This has an important application to scanning chiral surface to identify spatial distribution of handedness of chirality in high resolution, for example, at biomaterial surface or chiral-dependent reflective surface^25^.

In Fig. 6(f) is demonstrated a switching between positive and negative Y (channel 2) as the driving voltage (channel 1) is alternated between 1.25 *V* (Γ = 0.65) and 1.77 *V* (Γ = 0.35). Switching operation of post-selected optical spin Hall shift has a potential application to signal processing in nanoscale photonics.

In conclusion, the Berry connection and curvature are introduced to describe optical spin Hall shift in phase-discontinuity metasurface. A large refractive index gradient tangential to metasurface allows a sign change in relative transverse shift, upon varying incidence angle of an optical beam. By adopting a weak value measurement, it is demonstrated that optical spin Hall shift can be controlled by manipulating optical phase retardance in the post-selection. Furthermore, switching operation of post-selected optical spin Hall shift is shown as an example of dynamic control of transverse shift. Control of optical spin Hall shift in the optical far field has strong implication of applications where optical spin is utilized as a degree of freedom for signal processing, angular momentum transfer, sensing, and scanning chiral surface.

## Methods

### Sample fabrication

Phase-discontinuity metasurface is composed of V-shape antenna pattern^15^. A linear array of eight V-shape apertures is repeated along *x*-axis with the lattice constant Γ of 2400 nm. Focused ion beam milling is utilized to fabricate Babinet complementary V-shaped antennas on e-beam evaporated 30 nm-thick Au film on top of fused silica substrate with adhesion layer of 3 nm thick titanium^26^.

### Experimental set-up

We adopted 10 *mW λ* = 1310 *nm* pigtail style self-contained thermally stabilized laser diode as the light source (OZ optics-OZ-2000) with the output fiber diameter 50 *μ*m. The beam passes through a Glan/Thomson polarizer *P*1 (Thorlabs-GL10-C) to be linearly polarized. Then it is focused onto the metasurface with a microscope objective lens, *f* = 95 *mm*, to a 1/*e*^2^ intensity spot size *w*_0_ = 50*μm*. The extraordinary refraction beam is collected with a microscope objective lens, *f* = 95 *mm*, and a liquid crystal variable retarder (Thorlabs-LCC1113-C) and a second polarizer *P*2 are adopted to resolve the polarization state with an InGaAs-based NIR camera (Ophir-XC-130), and InGaAs-based quadrant position sensitive detector (Newport-2903) with a 3-mm diameter active region is employed for imaging and detection. In our experimental set-up, the propagation distance is *z* = *f* = 60 *z*_0_. Position sensitive detector is connected to the oscilloscope, and the position X, Y, and SUM data are monitored. The relative transverse shift 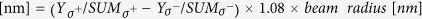, which is obtained from the light intensity measurement by a photo-reciver placed on two-dimensional translation stage.

## Additional Information

**How to cite this article**: Lee, Y.U. and Wu, J.W. Control of optical spin Hall shift in phase-discontinuity metasurface by weak value measurement post-selection. *Sci. Rep.* **5**, 13900; doi: 10.1038/srep13900 (2015).

## Supplementary Material

Supplementary Information

## Figures and Tables

**Figure 1:**
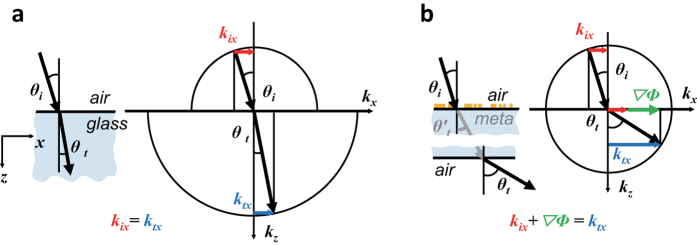
Schematics of optical beam refractions with phase gradient 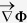 in the incidence plane. (**a**) At air-glass interface 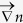 is along *z*-axis normal to the surface, and radii of circles of equifrequency contour are different in air and glass. (**b**) At phase-discontinuity metasurface 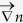 is along *x*-axis tangential to the surface, and the net refraction after passing through the glass substrate is described by circle of equifrequency contour in air with an additional momentum from 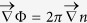.
