# Supplementary material for: Control of optical spin Hall shift in phase-discontinuity metasurface by weak value measurement post-selection
Source: Sci Rep. 2015 Sep 10;5:13900. doi: 10.1038/srep13900 (PMC4564808; doi:10.1038/srep13900)
Supplement: Supplementary Information [file srep13900-s1.pdf]

SUPPLEMENTARY INFORMATION

Control of optical spin Hall shift in phase-discontinuity metasurface by weak value measurement post-selection

Y.U. Lee, J.W. Wu*

Department of Physics and Quantum Metamaterials Research Center

Ewha Womans University, Seoul 120-750, Korea

* Email: jwwu@ewha.ac.kr

S1. Derivation of OSH shift in terms of Berry connections in PMS

The transverse shift upon refraction has been related to the Berry connection.[1,2]

$$\delta y = \langle z^i \left|\Lambda_{k^i}\right| z^i \rangle - \langle z^t \left|\Lambda_{k^t}\right| z^t \rangle \tag{S1}$$

where $|z^{t,i}\rangle$ and $\Lambda_{k^{t,i}}$ stand for polarization state and Berry connection of refraction and incidence beams. Bliokh *et al.* expressed the Berry connection $\hat{A}^{(\lambda)}$ in terms of rectangular components of the linear momentum to obtain OSH shift δy of optical beam with spin λ.[2] For PMS surface, we have

$$\begin{aligned}
\delta y &= \hat{A}^{(\lambda)}_{iy} - \hat{A}^{(\lambda)}_{ty} \\
&= \lambda \left(\frac{p_{tx}p_{tz}}{p_t(p_{tx}^2 + p_{ty}^2)} - \frac{p_{ix}p_{iz}}{p_i(p_{ix}^2 + p_{iy}^2)} \right) \\
&= \lambda \left(\frac{p_{tz}}{p_t p_{tx}} - \frac{p_{iz}}{p_i p_{ix}} \right) \\
&= -\lambda \frac{\cos\theta_t - \cos\theta_i}{|\nabla\Phi|}
\end{aligned} \tag{S2}$$

where $p_{tx} = -\hbar|\nabla\Phi|$, $p_{tz} = \hbar k_t \cos\theta_t$, $p_i = p_t = \hbar k_i = \hbar k_t$, $p_{ix} = -\hbar|\nabla\Phi|$, $p_{iz} = \hbar k_i \cos\theta_i$, $\nabla\Phi = -|\nabla\Phi|\hat{x}$, and it is noted that $p_{iy} = p_{ty} = 0$.

Reference

1. Onoda, M., Murakami, S. & Nagaosa, N. Geometrical aspects in optical wave-packet dynamics. *Phys. Rev. E* **74**, 066610 (2006).
2. Bliokh, K. Y. & Freilikher, V. Topological spin transport of photons: Magnetic monopole gauge field in maxwell's equations and polarization splitting of rays in periodically inhomogeneous media. *Phys. Rev. B* **72**, 035108 (2005).
